# Meta-Analysis and Systematic Review in Environmental Tobacco Smoke Risk of Female Lung Cancer by Research Type

**DOI:** 10.3390/ijerph15071348

**Published:** 2018-06-27

**Authors:** Xue Ni, Ning Xu, Qiang Wang

**Affiliations:** National Institute of Environmental Health, Chinese Center for Disease Control and Prevention, No. 29 Nanwei Road, Beijing 100050, China; nxup2016@163.com (X.N.); xn@nieh.chinacdc.cn (N.X.)

**Keywords:** Environmental Tobacco Smoke, risk, female lung cancer, meta-analysis, study type

## Abstract

More than 50% of women worldwide are exposed to Environmental Tobacco Smoke (ETS). The impact of ETS on lung cancer remains unclear. Cohort studies since the late 1990s have provided new evidence of female lung cancer risk due to ETS. The objective of this meta-analysis and systematic review was to analyze the association of ETS with female lung cancer risk from 1997 to 2017, organised based on research design. According to our applied inclusion and exclusion criteria, 41 published studies were included. The relative risk (RR) from the cohort studies or odds ratio (OR) from case-control studies were extracted to calculate the pooled risks based on the type of study. The summary risks of ETS were further explored with the modulators of ETS exposure sources and doses. The pooled risks of lung cancer in non-smoking women exposed to ETS were 1.35 (95% CI: 1.17–1.56), 1.17 (95% CI: 0.94–1.44), and 1.33 (95% CI: 1.17–1.51) for case-control studies, cohort studies, and both types of studies, respectively. The summary RR estimate of the cohort studies was not statistically significant, but the RR increased with increasing doses of ETS exposure (*p* trend < 0.05). Based on the results of this study, ETS might be an important risk factor of female lung cancer in non-smokers.

## 1. Introduction

Tobacco smoke has been proved to be the main factor influencing the risk of lung cancer. Except for carcinogens in the main stream of tobacco smoke, carcinogens such as benzo[a]pyrene, *N*′-nitrosonornicotine (NNN), and (methylnitrosamino)-1-(3-pyridyl)-1-butanone (NNK) are rich in sidestream smoke, also called second-hand smoke [1]. In addition, the pollutants in residual tobacco smoke absorbed by clothing, hair, furnishings, and dust are labelled as third-hand smoke that contribute as a secondary source of indoor Environmental Tobacco Smoke (ETS) [2,3]. Statistics about the global burden of disease related to ETS released by the World Health Organization (WHO) in 2011 showed that the global average proportion of children with at least one smoking parent, according to the definition from Global Youth Tobacco Survey, was estimated to be 41%, and the female adult ETS proportion was about 63% [4]. Such high prevalence of ETS is causing public health concerns. However, the association of ETS with the risk of lung cancer remains unclear.

Because interviews are convenient, most studies that focused on the relationship between ETS and lung cancer in non-smokers were case-control studies. Most of the case-control studies suggested that ETS might significantly increase lung cancer risk. However, limited to recall bias and relatively small sample size, the evidence provided in these case-control studies was relatively weak. The International Lung Cancer Consortium (ILCCO) pooled 18 case-control studies with pooling data in their databank in 2014 [5] and screened a total of 2504 non-smoking lung cancer patients. Their analysis showed a distinct association between ETS and lung cancer risk. After controlling for age, sex, race and ethnicity, and study, the adjusted odds ratio (OR) was 1.31 (95% confidence interval (CI): 1.17–1.47).

Due to the time, cost, and follow-up issues, the number of cohort studies specific to the association of female lung cancer risk in non-smokers with ETS exposure is quite small. Most of the cohort studies found no significant lung cancer risks [6,7,8,9,10,11,12]. In addition, several cohort studies, such as The Women’s Health Initiative Observational Study (WHI-OS) [13] and Shanghai Women’s Health Study [14], found that the ETS relative risk (RR) of non-smoking female lung cancer was less than 1.0 and the RR was not statistically significant.

Although many meta-analyses have been completed on the association of ETS with lung cancer, the meta-analyses were mostly conducted prior to 2010. Most previous meta-analyses were mixed analyses of female and male lung cancer risk [15,16,17,18,19] or analyzed single exposure sources [20,21,22]. In addition, many meta-analyses did not distinguish between the risk of lung cancer occurrence and the risk of death [15,22,23,24,25]. Although the differences in the types of studies were considered in two of the previous meta-analyses, possibly due to the fewer number of cohort studies, the findings from the two different types of studies were summarized together in a group [15,24]. To the best of our knowledge, cohort studies may have stronger evidence weights than case-control studies, so separately estimating the pooled risks of cohort studies from case-control studies may provide stronger results.

Considerable new evidence about the ETS risks of female lung cancer in non-smokers has emerged since the late 1990s. In particular, some cohort studies provided evidence of the risk of ETS and lung cancer. In order to further clarify the association of ETS with non-smoking female lung cancer risk, the goal of this meta-analysis was to estimate the summarized risk with studies published since 1997 based on the modulator of research type (cohort studies or case-control studies) and by the dose of ETS exposure.

## 2. Materials and Methods

### 2.1. Data Collection

We strictly followed the Preferred Reporting Items for Systematic Reviews and Meta-Analyses (PRISMA) requirements for literature retrieval and writing. The search terms ((“lung neoplasms” [MeSH Terms]) OR “lung cancer” [All Fields]) AND ((“tobacco smoke pollution” [MeSH Terms]) OR “passive smoking” [All Fields] OR “environmental tobacco smoke” [All Fields] OR “secondhand smoke” [All Fields]) AND (“1997/01/01” [PDAT]: “2017/12/31” [PDAT]) were used to search PubMed and (‘lung cancer’: ti,ab,kw OR ‘lung carcinoma’: ti,ab,kw) AND (‘environmental tobacco smoke’: ti,ab,kw OR ‘passive smoking’: ti,ab,kw) AND [1997–2017]/py were used as the search string for Embase. We collected the literature on female lung cancer and ETS published from 1997 to 2017. In addition, the references used in each study, the previous meta-analyses about the association of female lung cancer with ETS published publicly, and the reports about female lung cancer projects conducted in various countries were reviewed to select qualified literature.

### 2.2. Inclusion and Exclusion Criteria

We selected studies for the meta-analysis using the following inclusion criteria: (1) a cohort study or a case-control study about ETS and female lung cancer risk published between 1997 and 2017; (2) the number of participants was clearly described; (3) lung cancer cases diagnosed by physicians; (4) ETS clearly defined; and (5) OR, RR, or detailed data were provided.

We excluded studies using the following rules: (1) the ETS of female participants could not be distinguished distinctly, (2) the rules for participant enrollment were not clearly stated, (3) the study was only about genetic susceptibility of lung cancer, (4) the type of study was neither a cohort study nor a case-control study, (5) the same study was found repeatedly in other journals, (6) a subset of the same study was published elsewhere, (7) the score of quality assessment was relatively lower or the quality control of the study was not fully illustrated, and (8) the outcome of the study was death due to lung cancer.

### 2.3. Study Selection

**Figure 1:**
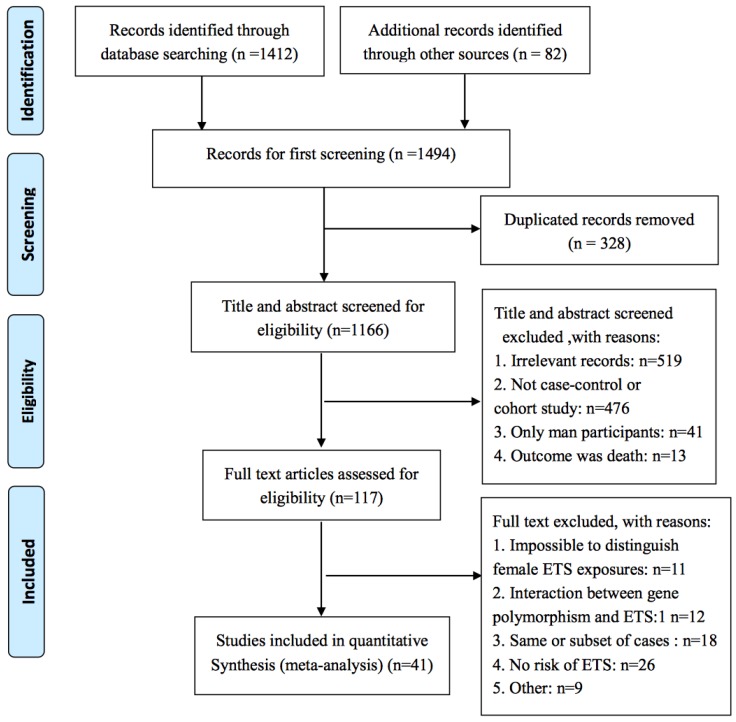
Preferred Reporting Items for Systematic Reviews and Meta-Analyses (PRISMA) literature screening flow diagram.

A total of 1494 published studies from 82 different sources were collected. After excluding duplicated records (*n* = 328), we screened studies by title and abstract and 117 studies remained according to our inclusion and exclusion criteria. We then read the full text of the remaining 117 studies. Studies that were impossible to distinguish female ETS exposure [26,27,28,29,30,31,32,33,34,35,36], studies without risk of ETS [37,38,39,40,41,42,43,44,45,46,47,48,49,50,51,52,53,54,55,56,57,58,59,60,61,62], studies that were published more than once, studies with a subset of cases [63,64,65,66,67,68,69,70,71,72,73,74,75,76,77,78,79,80], studies with lower quality scores (less than five points), and studies about the interaction between gene polymorphism and ETS [81,82,83,84,85,86,87,88,89,90,91,92] were excluded. In addition, the studies of Seow et al. [93], Edwards [94], and Chen et al. [95], due to the different definitions of ETS, were also excluded. The risk of ETS in studies completed by Schwartz et al. [96] and Ferreccio et al. [97] were reported only as an interactive item, so these two studies were also excluded. The screening process is shown in Figure 1.

### 2.4. Definition of ETS and Never Smoker

In this study, ETS was defined as self-reported exposures of never smokers who have been exposed to ETS at family or at workplace at any point in time. We further classified ETS into four categories based on exposure source. The categories of ETS exposures are listed in Table 1.

As for never smokers in this study, never smokers were defined as participants who never smoked or who had smoked less than 100 cigarettes in their lifetime.

### 2.5. Definition of ETS Exposure Dose

We classified the ETS dose into the following groups by pack-years, years of exposure, and cigarettes per day:

(1) If the ETS exposure was less than 20 pack-years, then the ETS exposure was defined as low pack-year, and if the ETS exposure was 20 or more pack-years then the ETS exposure was defined as high pack-year.
(2) If the ETS exposure was less than 20 years, then the ETS was defined as short-term ETS, and if the exposure was 20 or more years, then it was defined as long-term ETS.
(3) If the ETS exposure was less than 10 cigarettes per day, then the ETS was defined as light ETS, and if the ETS was 10 cigarettes or more, then it was defined as heavy ETS.

### 2.6. Quality Control

All studies were searched and screened by two authors with the same keywords and rules of literature selection. The literature qualities of case-control or cohort studies were evaluated according to The Newcastle-Ottawa Scale (NOS) [136]. Only studies with five or more points qualified for the meta-analysis. All results of OR, RR, and exposed/unexposed counts of ETS were double entered. In addition, we further proofread the data to ensure accuracy. All studies selected included the ETS risks of female lung cancer.

### 2.7. Statistical Analysis

The R version 3.4.2 metafor package was used for meta-analysis. OR, RR, or the detailed exposed and unexposed counts were selected as the indicators to estimate the pooled risks and to produce the forest plot. The Q statistic and the I^2^ index were used to determine the heterogeneity of the studies [137]. Publication bias was tested by funnel plot and Egger’s linear regression method [138]. In addition, sensitivity analysis and publication bias were checked using “trim and fill” non-parametric trimming methods.

## 3. Results

### 3.1. Characteristics of Included Studies

A total of 41 studies were included based on the inclusion and exclusion criteria, including seven cohort studies and 34 case-control studies. Among them, 26 were conducted in Asia, 8 in Europe, and 7 in North America. Meta-analysis showed some heterogeneity in all included studies (I^2^ = 67%, 95% UI: 55–76%). The random effect model was used to analyze the pooled effect and the pooled RR was 1.33 (95% CI: 1.17–1.51). In 8 of the 41 studies, the lung cancer risk of ETS exposure was less than 1.0 and only the OR of one study (Neuberger [120]) found a negative correlation that was statistically significant. The other studies showed the risk increased (OR/RR > 1.0).

### 3.2. Association of ETS with Female Lung Cancer by Different Study Type

We found that the heterogeneity of all studies was relatively large, so we stratified all studies according to the type of study. As a result, the pooled RR of seven cohort studies was 1.17 (95% CI: 0.94–1.44). The RRs in five of the seven studies were larger than 1.0 (RR = 1.2–1.9), and the RR of only one study was statistically significant. The RR in two studies showed that ETS and female lung cancer was negatively associated (RR < 1.0). As to the 34 case-control studies, the pooled OR was 1.35 (95% CI: 1.17–1.56). Of the 34 studies, 29 were positively related to lung cancer (RR > 1.0) and 10 were statistically significant (*p* < 0.05). Table 2 and Table 3 list the summary risks of all studies based on the type of study. As for the confounding effects, meta-regression results showed that there was no statistically significant difference between the adjusted and unadjusted risks (*p* = 0.59).

### 3.3. Association of Female Lung Cancer with ETS Based on Exposure Source

Due to the different ETS risks in two types of studies, we estimated the pooled risks of exposure source based on the strata of the type of study. Table 4 shows that the pooled risk for ETS from the workplace was higher than that from family in both cohort and case-control studies. In addition, we found the pooled OR for ETS from both family and workplace was significantly higher than from any single ETS source in the case-control studies (*p* < 0.05). However, this association was not found in cohort studies.

### 3.4. Association of Female Lung Cancer with ETS Based on Different Exposure Dose

#### 3.4.1. Association of Female Lung Cancer with ETS Exposure Dose in Cohort Studies

According to the exposure doses described in studies, the ETS dose was recorded using one of three methods: pack-year, duration (exposure year), and cigarettes per day. Due to the different definitions of ETS dose in the qualified cohort studies, there were no more than three studies for each category. So this part was not fit for meta-analysis to summarize the RR by ETS dose. Thus, we estimated the dose-response trend and found that the RR significantly increased with increasing dose in four of five studies (Table 5).

#### 3.4.2. Association of Female Lung Cancer with ETS Exposure Dose in Case-Control Studies

The pooled risk of ETS dose by pack-years, years of exposure, and cigarettes per day was summarized. Table 6 shows the stratified meta-analysis results of ETS by dose. Stratified by the pack-year of ETS exposure, the meta-analysis showed that the risk of high pack-year was significantly higher than that of low pack-year (*p* < 0.05). As for studies with ETS exposure dose described by exposure years, risk of long-term ETS (≥20 years) was not found to be higher than that with short-term, ETS (<20 years) by stratified meta-analysis. As for the studies with ETS exposure dose described by cigarettes per day, stratified meta-analysis showed that neither light ETS (<10 cigarettes per day) nor heavy ETS (≥10 cigarettes per day) was significantly associated with increased risk of lung cancer (*p* > 0.05).

### 3.5. Bias of Publications

**Figure 2:**
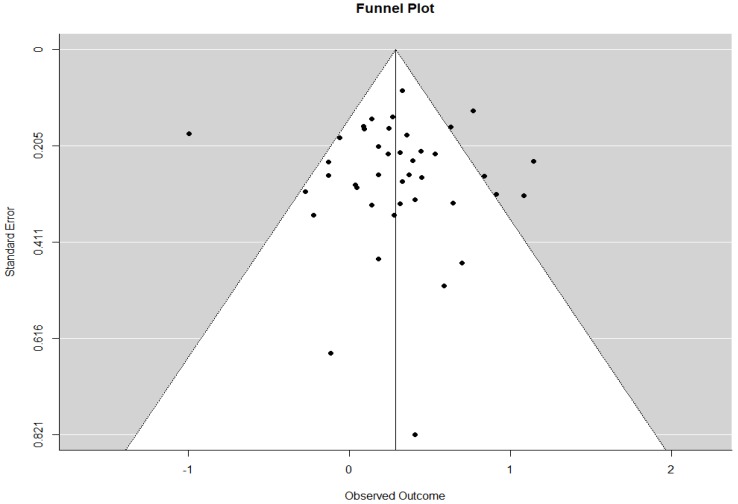
Funnel plot of the association of ETS with female lung cancer.

Publication bias was examined by funnel plot and Egger’s test. The publication bias was relatively small. The funnel plot was symmetrical (Figure 2), and the Egger’s test was not significant (*z* = 0.42, *p* = 0.67). When the trim and fill algorithm was used to calibrate the result, the estimated number of missing studies on the right side was zero.

### 3.6. Heterogeneity

The heterogeneity of the seven included cohort studies was small (I^2^ = 2%, 95% UI: 0–24%); however, the heterogeneity of the case-control studies in our meta-analysis was relatively large (I^2^ = 71%, 95% UI: 59–80%). The study of Neuberger [120] considerably contributed to the heterogeneity. After removing the study of Neuberger [120], further sensitivity analysis found that I^2^ dropped to 49%. Table 4 lists the heterogeneity by type of studies and sources of ETS in detail.

### 3.7. Previous Meta-Analyses

A total of 31 meta-analyses were published about the lung cancer risk of ETS: 24 meta-analyses were about the ETS risks from spouses, 11 were about the ETS risks from the workplace, and 7 were about the ETS risks of childhood exposure. As to ETS exposures from spouses, 22 of 24 studies suggested significantly increased risks of ETS. As for workplace ETS exposures, 9 of 11 studies suggested significantly increased risks of ETS. As to childhood ETS exposure, only two studies suggested significantly increased risks. The number of ETS studies included in the previous meta-analyses were different. The summarized risks of ETS varied by meta-analysis; however, the pooled risks in 21 of 31 meta-analyses were in the range of 1.2 to 1.4. Table 7 lists the 31 previous meta-analyses in detail.

## 4. Discussion

We retrieved studies on the association of female lung cancer with ETS from 1997 to 2017. We found a weak association of ETS with female lung cancer, with the risk of lung cancer increasing by about 33%. Our result was similar to that of the International Agency for Research on Cancer (IARC) ETS assessment in 2012. However, we found the association was inconsistent between cohort and case-control studies. We tried to compare our results with those of previous published meta-analyses but all 31 meta-analyses did not distinguish the outcome (death or newly developed cases) in their pooled estimates. In only two of the meta-analyses were the pooled risks of ETS summarized by type of study (Taylor [22], Zhong [23]). However, the two meta-analyses did not limit the outcome to death or new case. The pooled risk of ETS in Taylor’s meta-analysis [22] for case-control studies was similar with our result, but the pooled risks of the cohort studies of the two meta-analyses were somewhat different from our result. The pooled RRs in their meta-analyses suggested a significantly increased risk of lung cancer for ETS exposure. The pooled RR estimate in our meta-analysis was similar to that of Taylor [22], but the pooled risk was not significant due to the two new cohort studies included.

Based on the difference in pooled risks between cohort and case-control studies, we further analyzed the association between female lung cancer and ETS source or ETS dose based on type of study. First, for risks based on exposure source, we did not find a higher risk of exposure to multiple sources than from a single source of ETS in cohort studies, but we found an association in case-control studies. Due to the availability of uniformed ETS doses, comparing multiple ETS exposure doses to single ETS exposures or to sum ETS doses by different source for different designs of previously published studies was not feasible. Thus, the risk of multiple ETS sources needs to be validated in subsequent large sample studies. This is also one of the uncertainties in our analysis. As to the risk of ETS doses, we found a consistency in dose trend in both cohort and case-control studies. We observed a significant trend in the exposure doses of ETS in five cohort studies. In case-control studies, we found dose trends in pack-year or cigarettes per day; the dose trend of pack-year was statistically significant.

Based on the results of Egger’s test and the trim and fill algorithm, the inclusion of the studies was relatively reasonable. The Egger’s test was not statistically significant and the pooled risk was not updated after the calibration of the trim and fill algorithm. All the studies included in this meta-analysis were papers in peer reviewed journals and therefore some publication bias may exist. Fortunately, the number of unpublished studies of ETS and lung cancer was quite small [160].

In addition, seven studies in our meta-analysis reported the risks of multiple ETS exposure sources. Pooling the risks of the seven studies was impossible due to lack of counts of exposed and unexposed of single-specified ETS sources. For these seven studies, we chose the smallest OR of all sources as the inputs for our meta-analysis. This may have been one of the sources of heterogeneity. As a result, the pooled risk in our study is relatively conservative.

Although the association of ETS with lung cancer has been studied for a long time, the exposure classification is not uniform in all studies and various designs have been used for ETS exposure assessments. Fewer studies assessed ETS exposure based on exposure biomarkers for internal doses. To the best of our knowledge, previous studies showed that the association of ETS with lung cancer risk was relatively weak and the association might be confounded. Aging is one of the known confounders of lung cancer. So, age was adjusted in most studies for the ETS risk of lung cancer. Our meta-regression results showed that regardless of whether age was adjusted, the summary risks of ETS did not change significantly. We hypothesize that the reason for the minimal difference between crude risks and the adjusted risks is that the confounding effects of age might be relatively weaker. Apart from age, the common confounding factors of the ETS risk of lung cancer include air pollution, low fruit intake, radon exposure, and some occupational carcinogens. However, the limited difference may also be due to the difficulty of obtaining data or the smaller risks of some factors. Few studies have controlled these factors when estimating the adjusted risk of ETS exposure. Whether the ETS risk of lung cancer is significantly confounded by the above-mentioned risks must to be further explored in the future studies.

Furthermore, the proportion of pathological diagnosis for lung cancer cases in these studies was quite low and the misclassification of cases cannot be ignored. All these factors might limit the association of ETS with female lung cancer, which creates uncertainties in this study.

## 5. Conclusions

In summary, the results of case-control studies and cohort studies were not consistent, but the dose trends in the association of ETS with female lung cancer indicated that heavy exposures of ETS, especially for ETS exposures for more than 20 years, is the dominant determinant of lung cancer risk of ETS, irrespective of type of study. Due to the high proportion of ETS worldwide, the impact of ETS on female lung cancer is an important public health concern.

## Figures and Tables

**Table 1:** Categories of Environmental Tobacco Smoke (ETS) exposures.

ETS Category	Definition	References
Workplace ETS	ETS from smoking colleagues who worked in the same office or workplace	[13,98,99,100,101,102,103,104,105,106,107,108,109,110,111]
Family ETS	ETS from parents in childhood, husbands of current smokers or ever smokers, or other family smokers	[11,13,98,99,100,101,102,103,104,105,106,107,108,109,110,111,112,113,114,115,116,117,118,119,120,121,122,123]
Family and Workplace ETS	ETS both from family and workplace	[13,98,102,103,109,114,117,121,124]
Unknown ETS	ETS source was not specified	[13,14,99,101,102,103,105,107,108,109,113,114,115,117,121,124,125,126,127,128,129,130,131,132,133,134,135]

**Table 2:** Meta-analysis of the association of female lung cancer with ETS in cohort studies.

Author	Year	Country	RR	95% CI	Adjustment
Jee et al. [112]	1999	Korea	1.90	1.00–3.50	Yes: age, socioeconomic status, residency, vegetable consumption, occupation
Speize et al. [125]	1999	U.S.	1.50	0.30–6.30	Yes: age
Nishino et al. [11]	2001	Japan	1.80	0.67–4.60	Yes: age, study area, alcohol, diet, history of lung diseases
Vineis et al. [126]	2005	Europe	1.20	0.71–2.02	Yes: age, sex, smoking, country, school years
Weiss et al. [14]	2008	China	0.94	0.65–1.35	No
Kurahashi et al. [98]	2008	Japan	1.45	0.86–2.44	No
Wang et al. [13]	2015	U.S.	0.88	0.52–1.49	Yes: age, body mass index (BMI), ethnicity, history of lung cancer, family history of cancer, education, occupation, hormone therapy use, oral contraceptive use, fruit servings per day, vegetable servings per day, red meat serving per day, alcohol, physical activity
Pooled RR (Fixed effect) RR: 1.17, 95% CI: 0.94–1.44					

**Table 3:** Meta-analysis of the association of female lung cancer with ETS in case-control studies.

Author	Year	Country	OR	95% CI	Adjustment
Zheng et al. [129]	1997	China	1.04	0.59–1.85	No
Ko ^1^ et al. [104]	1997	Taiwan	0.80	0.40–1.60	Yes: socioeconomic status, residential area, education
Dai et al. [123]	1997	China	3.14	1.97–5.01	No
Boffetta et al. [108]	1998	Europe	1.15	0.86–1.55	Yes: age, sex
Nyberg ^1^ et al. [111]	1998	Sweden	0.76	0.42–1.37	Yes: age, gender, catchment area, occasional smoking, vegetable consumption, degree of urban residence, years of exposure to risk occupation
Song et al. [127]	1999	China	2.31	1.36–3.90	No
Zhong et al. [103]	1999	China	1.20	0.80–1.80	Yes: age, income, intake of vitamin C, respondent status, smokiness of cooking, family history of lung cancer, occupation
Zaridze ^1^ et al. [106]	1999	Russia	0.88	0.55–1.41	Yes: age, education
Rapiti ^1^ et al. [116]	1999	India	1.20	0.50–2.90	Yes: age, residence, religion
Zhou ^1^ et al. [100]	2000	China	0.89	0.25–3.16	No
Wang et al. [115]	2000	China	1.15	0.60–2.10	No
Lee et al. [105]	2000	Taiwan	1.88	1.36–2.60	No
Kreuzer et al. [107]	2000	Germany	1.09	0.79–1.50	No
Johnson et al. [117]	2001	Canada	1.32	0.66–2.63	No
Fang et al. [99]	2002	China	2.95	1.60–5.47	No
Rachtan [118]	2002	Poland	2.49	1.36–4.54	Yes: age, diet, siblings with cancer, tuberculosis, place of residence, occupational exposure, pack-years smoking
Kubík et al. [121]	2002	Czech	1.05	0.59–1.86	No
Chan-Yeung et al. [114]	2003	Hong Kong	1.57	0.92–2.68	No
Phukan et al. [135]	2005	India	1.56	1.02–2.39	Yes: age, education, occupational status
Yu et al. [124]	2006	Hong Kong	1.39	0.80–2.41	No
Francomarina et al. [119]	2006	Mexico	1.70	1.10–2.80	Yes: age, educational level, access to social security
Neuberger ^1^ et al. [120]	2006	U.S.	0.37	0.26–0.54	No
Gorlova et al. [109]	2006	U.S.	1.27	0.82–1.97	No
Rylander ^1^ et al. [110]	2006	Sweden	1.37	0.72–2.61	No
Liang et al. [113]	2009	China	1.43	1.00–2.07	Yes: age, marital status, years of schooling, ethnicity, BMI, 5 years ago
Hosseini et al. [132]	2009	Iran	1.50	0.80–3.00	No
Mu et al. [101]	2013	China	1.48	0.93–2.35	No
Lo et al. [102]	2013	Taiwan	1.39	1.17–1.67	Yes: age, years of education
Seki et al. [122]	2013	Japan	1.31	0.99–1.72	Yes: age, year of recruitment, area of residence, referral status, occupation, alcohol drinking, family history of lung cancer
Yin et al. [130]	2014	China	1.28	0.92–1.79	Yes: age
Behera et al. [134]	2014	India	2.01	0.83–4.92	Yes: smoking, cooking fuel, residence, occupational history
Kim et al. [131]	2015	U.S.	1.37	0.89–2.10	Yes: age, sex, race/ethnicity
Ren et al. [133]	2015	China	1.10	0.79–1.53	No
He et al. [128]	2017	China	2.16	1.67–2.80	No
Pooled OR (Random effect) OR: 1.35, 95% CI: 1.17–1.56					

^1^ Study contained a variety of ETS exposure sources. The risks of all ETS sources could not be pooled with the data reported in the study, so the smallest OR of various ETS risks in the study was used in the meta-analysis.

**Table 4:** Meta-analysis of ETS exposure source and female lung cancer.

Exposure Source	Number	RR (95% CI)	I^2^ (95% UI)	*p* Value	Model
Cohort Studies					
Family	4	1.40 (1.08–1.82)	0 (0–85)	0.61	Fixed
Workplace	2	1.54 (0.61–3.91)	74 (0–94)	0.05	Random
Family and Workplace	2	1.10 (0.71–1.69)	55 (0–89)	0.14	Fixed
Unknown	4	0.99 (0.77–1.29)	0 (0–79)	0.79	Fixed
Case-Control Studies					
Family	24	1.27 (1.05–1.53)	75 (64–83)	<0.01	Random
Workplace	13	1.36 (1.21–1.53)	37 (0–67)	0.09	Fixed
Family and Workplace	7	1.75 (1.43–2.14)	0 (0–61)	0.05	Random
Unknown	23	1.43 (1.32–1.55)	38 (0–71)	0.86	Fixed

**Table 5:** Overview of the association of ETS dose with female lung cancer in cohort studies.

Exposure	Study	Exposure Categories	RR (95% CI)	*p* Trend
Pack-year ^1^	Kurahashi	<30	1.05 (0.55–2.02)	0.03
		≥30	1.46 (0.85–2.50)	
Duration(year)	Wang	<20	1.11 (0.74–1.65)	0.24
		20–30	1.11 (0.63–1.96)	
		≥30	1.61 (1.00–2.58)	
	Jee	1–29	1.60 (0.80–3.00)	<0.01
		≥30	3.10 (1.40–6.60)	
Cigarettes/day	Kurahashi	<20	1.02 (0.51–2.04)	0.02
		≥20	1.47 (0.87–2.49)	
	Jee	1–19	2.00 (1.10–3.90)	<0.10
		≥20	1.50 (0.70–3.30)	

Pack-year ^1^ = cigarettes smoked every day/20 × smoking year.

**Table 6:** Stratified meta-analysis of dose association of Environmental Tobacco Smoke (ETS) with female lung cancer in case-control studies.

Exposures	Number	OR (95% CI)	I^2^ (95% UI)	*p* Value	Model
Pack-Year					
<20	4	0.93 (0.77–1.13)	25 (0–71)	0.26	Fixed
≥20	3	1.74 (1.04–2.90)	74 (11–92)	0.02	Random
Years of Exposure					
<20	7	1.71 (1.01–2.90)	79 (58–90)	<0.01	Random
≥20	6	1.57 (1.05–2.35)	70 (31–87)	<0.01	Random
Cigarettes/Day					
<10	4	1.23 (0.90–1.69)	61 (0–87)	0.05	Random
≥10	4	1.53 (0.69–3.40)	88 (72–95)	<0.01	Random

**Table 7:** Published meta-analyses of ETS and lung cancer.

ID	Author	Number of Studies	Sex	Pooled OR or RR (95% CI)	Exposure Source
1	Boffetta et al. [139]	45	F	1.25 (1.14–1.38)	Spouse
		15	F	1.17 (1.02–1.33)	Work
2	Taylor et al. [22]	43	F	1.29 (1.17–1.43)	Spouse
3	Lee et al. [15]	93	F	1.22 (1.14–1.31)	Spouse
		47	F and M	1.22 (1.15–1.30)	Work
		41	F and M	1.15 (1.02–1.29)	Childhood
4	Zhong et al. [23]	40	F	1.20 (1.12–1.29)	Spouse
		14	F	1.15 (1.04–1.28)	Work
		18	F	0.89 (0.81–0.98)	Childhood
5	Hackshaw et al. [140]	37	F	1.24 (1.13–1.36)	Spouse
6	Taylor et al. [25]	55	F	1.27 (1.17–1.37)	Spouse
7	Gross [141]	31	F	1.18 (1.06–1.28)	Spouse
8	Wang [21]	6	F	0.91 (0.75–1.10)	Spouse
9	Tweedie et al. [142]	36	F	1.22 (1.08–1.37)	Spouse
		9	F	1.10 (0.90–1.32)	Work
10	U.S. National Research Council [143]	13	F	1.32 (1.16–1.52)	Spouse
11	Blot et al. [144]	12	F	1.30 (1.10–1.50)	Spouse
12	Wells [145]	17	F	1.44 (1.26–1.66)	Spouse
13	Lee [146]	28	F	1.18 (1.07–1.30)	Spouse
14	U.S. Environmental Protective Agency [147]	11	F	1.19 (1.01–1.39)	Spouse
15	Pershagen [148]	25	F	1.23 (1.11–1.36)	Spouse
16	Mengersen et al. [149]	34	F	1.23 (1.08–1.41)	Spouse
17	Dockery [150]	33	F	1.27 (1.18–1.38)	Spouse
18	Zhao et al. [151]	4	F and M	1.18 (0.80–1.74)	Spouse
		5	F and M	1.41 (1.19–1.66)	Work
		3	F and M	1.04 (0.86–1.27)	Childhood
19	Wald et al. [152]	13	F and M	1.35 (1.20–1.53)	Spouse
20	Saracci et al. [153]	14	F and M	1.35 (1.20–1.53)	Spouse
21	Law et al. [154]	34	F and M	1.24 (1.11–1.38)	Spouse
22	Merletti et al. [18]	39	F and M	1.24 (1.15–1.34)	Spouse
23	Tweedie et al. [155]	26	F	1.17 (1.06–1.28)	Spouse
24	Li et al. [156]	5	F and M	1.15 (1.00–1.33)	Spouse
		2	F and M	1.21 (1.09–1.34)	Childhood
25	Yu et al. [157]	8	F	1.47 (1.28–1.69)	Work
		8	F	0.99 (0.85–1.15)	Childhood
26	Stayner et al. [19]	22	F and M	1.24 (1.18–1.29)	Work
27	Fu et al. [158]	12	F and M	1.38 (1.13–1.69)	Work
		5	F and M	1.37 (0.98–1.91)	Childhood
28	Wells [16]	5	F and M	1.39 (1.15–1.68)	Work
29	Brown et al. [20]	14	F	1.25 (1.08–1.41)	Work
30	Levois et al. [159]	12	F and M	1.01 (0.92–1.11)	Work
31	Boffetta et al. [17]	10	F and M	0.91 (0.80–1.05)	Childhood
